# Association Between Isometric Hip Muscle Strength and Y-Balance Test Performance in Healthy Adults

**DOI:** 10.3390/jcm15135170

**Published:** 2026-07-02

**Authors:** Dragana Rasic, Kristijan Zulle, Bojan Miletic, Hrvoje Vlahovic

**Affiliations:** 1 Faculty of Health Studies, University of Mostar, 88000 Mostar, Bosnia and Herzegovina; dragana.rasic@fzs.sum.ba; 2 Faculty of Health Studies, University of Rijeka, 51000 Rijeka, Croatia; kristijan.zulle@fzsri.uniri.hr (K.Z.);

**Keywords:** dynamic balance, handheld dynamometry, hip muscle strength, Y-balance test

## Abstract

**Background/Objectives**: Dynamic balance during the Y-Balance Test (YBT) relies on coordinated multi-joint control of the lower extremity. Although hip muscle strength is considered important for YBT performance, the relative contribution of individual hip muscle groups remains insufficiently understood. This study aimed to examine the associations between isometric hip abduction, external rotation, and extension strength and YBT performance in healthy adults. **Methods**: In this cross-sectional study, 104 healthy adults underwent assessment of isometric hip abduction, external rotation, and extension strength using strap-stabilized handheld dynamometry. Hip extension strength was measured in the prone position with the knee flexed to 90°. Strength values were normalized and expressed as joint torque (Nm/kg). YBT performance was assessed in the anterior, posteromedial, and posterolateral directions and as a composite score. Associations were examined using Pearson correlation coefficients. To account for the dependency of bilateral measurements, a linear mixed model (LMM) was used to evaluate the collective and independent contribution of hip strength components to YBT performance, with sex, age, and BMI included as covariates. **Results**: All hip strength measures showed significant positive correlations with YBT performance (r = 0.19–0.49, *p* < 0.05). Hip extension strength demonstrated the strongest associations, particularly with posterolateral reach (r = 0.49). After adjustment for demographic covariates, sex was the strongest predictor of YBT performance across all directions (β = 7.8–8.9, *p* < 0.001), with males achieving higher scores than females. Hip extension and abduction strength were significant predictors of posterolateral reach (*p* < 0.05), whereas no hip strength variable independently predicted anterior reach or composite score after adjustment for demographic factors. No significant differences in YBT performance were observed between limbs. **Conclusions**: Sex was the strongest predictor of YBT performance in healthy adults. Hip extension and abduction strength were independently associated with posterolateral reach performance after controlling for demographic factors, suggesting that the association between hip muscle strength and dynamic balance may be direction-specific. These findings highlight the importance of accounting for sex when interpreting the relationship between hip strength and YBT performance.

## 1. Introduction

Dynamic balance and movement control represent key components of functional stability of the lower extremities and play an important role in injury prevention and the effectiveness of rehabilitation programs [1]. One of the most commonly used tests for assessing dynamic balance is the Y-Balance Test (YBT), a standardized derivative of the Star Excursion Balance Test (SEBT), which measures maximal reach distances in the anterior, posteromedial, and posterolateral directions, normalized to lower limb length [2,3]. Reduced reach distances, particularly in the anterior direction, have been associated with a higher risk of lower limb injuries, while asymmetries in reach performance are used to assess functional stability and the effectiveness of rehabilitation [4,5,6].

### 1.1. The Role of Hip Muscles in YBT

Stability and postural control in tasks such as the YBT depend on the integration of multiple muscle groups, with the hip muscles playing a key role in maintaining balance, controlling pelvic alignment, and stabilizing the center of mass [7,8]. The hip abductors, extensors, and external rotators contribute to lateral and rotational stability, enabling efficient control of lower limb movements. Weakness in these muscles has been associated with poorer YBT performance and an increased risk of injury [9,10]. Body composition, flexibility, and core stability also contribute to balance performance, and rehabilitation programs focused on strengthening the gluteal and core muscles have been associated with improved YBT scores [4].

### 1.2. Inconsistencies in the Literature and the Research Gap

Previous research has not been entirely consistent regarding which hip muscle groups most strongly influence YBT performance. While Wilson et al. identified hip abduction strength as the most important predictor, recent studies have reported that hip extension strength and ankle joint mobility also play significant roles in dynamic balance [8,11,12]. Domínguez-Navarro et al. indicated that hip adductors may also contribute to balance, whereas Nelson et al. highlighted that trunk motion and ground reaction forces significantly influence YBT performance independently of hip strength [13,14]. Some researchers have confirmed that hip strength is important, but not the sole factor contributing to balance control [15,16]. Importantly, most prior studies have examined clinical or athletic populations, including individuals with chronic ankle instability, anterior cruciate ligament reconstruction, or competitive athletes, in whom injury history and training adaptations may confound the relationship between hip strength and balance [17,18,19,20]. Establishing associations in a healthy, uninjured adult population therefore provides a necessary reference framework against which clinical deviations can be interpreted.

### 1.3. Research Justification, Purpose and Hypothesis

Despite growing interest in the relationship between hip strength and YBT performance, several important gaps remain in the existing literature. Few studies have simultaneously examined multiple hip muscle groups; abduction, external rotation, and extension, within the same healthy adult sample, making it difficult to determine the relative contribution of each muscle group to directional YBT performance. Additionally, sex has rarely been included in analyses of hip strength–balance relationships [11,21].

Understanding which hip muscle groups are most strongly associated with YBT performance may contribute to the identification of modifiable physical capacities relevant to injury screening and prevention programme design in the general population. Furthermore, determining whether the association between hip strength and balance is direction-specific could inform more targeted intervention strategies. Therefore, the aim of this study was to examine associations between isometric hip abduction, external rotation, and extension strength and YBT performance in healthy adults, and to identify which hip muscle components independently predict YBT outcomes. We hypothesized that all three hip strength measures would demonstrate significant positive associations with YBT performance, and that hip extension strength would show the strongest independent associations, particularly in the posterior reach directions.

## 2. Materials and Methods

### 2.1. Study Design

This cross-sectional study was conducted at the Faculty of Health Studies, University of Mostar, Bosnia and Herzegovina, from June to August 2025. The study followed the Declaration of Helsinki, and ethical approval was obtained from the Ethics Committee of the Faculty of Health Studies, University of Mostar (Approval No. 01-531/25; dated 5 May 2025). All participants provided written informed consent before participation. The study examined associations between isometric hip muscle strength and dynamic balance performance in a single testing session, with no intervention or follow-up assessment.

### 2.2. Participants

A total of 104 healthy adults (74% women, 26% men) volunteered to participate following a public recruitment call distributed through local media. Inclusion criteria were age 18–65 years, absence of acute lower-limb injury, no chronic conditions affecting movement, ability to perform all tests independently, and no trunk, hip, or knee injury within the previous 3 months. Exclusion criteria included musculoskeletal pain, neurological disorders, major postural deformities, or any condition that could impair test performance. Leg dominance was self-reported as the leg used for kicking a ball.

### 2.3. Procedure and Materials

All testing sessions were performed indoors under controlled environmental conditions, and participants completed all assessments during a single session. All assessments were conducted following a standardized warm-up and test demonstration. Participants wore sports clothing and completed testing in a fixed order (strength assessment → YBT) with standardized rest intervals to minimize fatigue. All measurements were performed by the same experienced examiner using standardized procedures.

#### 2.3.1. Isometric Hip Muscle Strength Assessment

Isometric hip muscle strength was assessed using a strap-stabilized handheld dynamometry system (Kinvent K-Push and K-Pull; Kinvent, Montpellier, France) connected to the Kinvent Physio App. Handheld dynamometry systems of this type have demonstrated acceptable reliability and validity across multiple muscle groups in previous research [22,23,24]; device-specific validation for hip measurements is discussed in the Limitations section. All measurements were performed by a single experienced examiner following standardized testing procedures. Hip abduction strength was measured in the supine position with the tested limb in neutral alignment. The dynamometer was positioned proximal to the lateral malleolus, and the pelvis was manually stabilized to minimize compensatory movements. Hip external rotation strength was assessed in a seated position with the hip and knee flexed to 90°. The dynamometer was placed against the medial aspect of the distal tibia, and participants were instructed to rotate the hip externally while maintaining an upright trunk posture. Hip extension strength was measured in the prone position with the knee flexed to 90° to minimize hamstring contribution and emphasize activation of the gluteus maximus. The dynamometer was positioned against the posterior aspect of the distal upper leg, and the pelvis was stabilized to prevent lumbar extension or pelvic rotation. Participants were instructed to extend the hip by pushing the thigh upward against the dynamometer while maintaining a neutral lumbopelvic position. For each muscle group, participants performed one familiarization trial followed by three maximal voluntary isometric contractions lasting 4 s, with 10 s rest intervals between trials. The highest force value across the trials was retained for analysis, consistent with standard practice in isometric strength testing, which assumes that the peak value best represents maximal voluntary muscle activation and minimises the influence of motivational variability across trials [25,26]. Peak force values (kg) were converted to joint torque (Nm) by multiplying force by the estimated lever-arm length, calculated as a standardized proportion of shank length based on anthropometric models [27,28].

Torque values were subsequently normalized to body mass (Nm/kg) to allow inter-individual comparison. Intrarater reliability for all strength measures was excellent (ICC(2,k) = 0.94–0.97) [27].

#### 2.3.2. Y-Balance Test Assessment

Dynamic balance was assessed using the standardized Y-Balance Test (YBT) [1,29,30]. Participants performed the test barefoot while standing on one leg at the center platform and reaching with the contralateral leg in the anterior, posteromedial, and posterolateral directions. One practice trial and three recorded trials were completed for each direction. Reach distances were measured in centimeters and normalized to leg length according to the following formula:


Normalized reach (%) = (reach distance/leg length) × 100

The composite score was calculated as:


Composite score = (ANT + PM + PL)/(3 × leg length) × 100

Trials were repeated in cases of balance loss or improper return to the starting position. Rest intervals of 10–15 s between trials and at least 30 s between directions were provided to minimize fatigue.

#### 2.3.3. Variables and Bias Control

Outcome variables included normalized YBT anterior, posteromedial, and posterolateral reach distances, as well as the composite score. Predictor variables included normalized isometric hip abduction, external rotation, and extension torque (Nm/kg). Age, sex, and BMI were included as covariates in the mixed-model analyses due to their established associations with muscle strength and balance performance.

Several strategies were implemented to reduce measurement bias. All assessments were conducted by the same trained examiner using identical instructions and demonstrations. A fixed testing order, standardized rest intervals, consistent equipment setup, and identical verbal encouragement during strength testing were applied across all participants.

Sample size estimation was performed using G*Power 3.1 [31].

Based on a moderate effect size (f^2^ = 0.15) derived from previous research examining the association between hip strength and YBT performance [8,9], a minimum of 92 participants was required to achieve 80% statistical power in a linear multiple regression model including up to five predictors, with a two-tailed α level of 0.05. The final sample of 104 participants exceeded this requirement, confirming that the study was adequately powered for all planned analyses. It should be noted that the power analysis was based on a standard regression framework; the linear mixed model approach used in the primary analyses may differ slightly in effective power due to the modelling of within-participant dependency, though the sample size is considered sufficient given the observed effect sizes.

### 2.4. Statistical Analysis

The normality of data distribution was assessed using the Shapiro–Wilk test. All study variables showed normal distribution (all *p* > 0.05), supporting the use of parametric statistical methods. Continuous variables are presented as means and standard deviations, and categorical variables as frequencies and percentages. The required sample size was determined as described in the Participants section. All statistical tests were two-tailed, with statistical significance set at *p* < 0.05.

To assess differences in YBT performance between the dominant and non-dominant limb, paired-samples t-tests were used. Since no statistically significant interlimb differences in YBT performance were observed, leg dominance was not included as a covariate in subsequent analyses. Both limbs were included in the analyses to increase statistical power and sample representativeness, with ipsilateral pairing of YBT and hip strength values (left YBT with left hip strength, right YBT with right hip strength). This approach is methodologically preferable to using a single limb, as it utilizes the full dataset while explicitly modeling the within-participant dependency of bilateral measurements, thereby reducing the risk of confounding due to arbitrary limb selection. Bivariate associations between hip strength measures and YBT outcomes were examined using Pearson’s correlation coefficient. Correlation strength was interpreted according to Mukaka: negligible (r < 0.30), low (0.30 ≤ r < 0.50), moderate (0.50 ≤ r < 0.70), high (0.70 ≤ r < 0.90), and very high (r ≥ 0.90) [32].

Bivariate correlations were computed across both limbs to provide descriptive estimates of association. The dependency of bilateral observations within participants was not corrected in the correlation analyses and should be interpreted accordingly.

To assess the collective and independent contributions of hip strength components to YBT performance while accounting for the dependency of bilateral measurements, linear mixed models (LMM) were applied with participants as a random effect. Hip abduction, external rotation, and extension torque were entered as fixed predictors, with sex, age, and BMI as covariates. Age and BMI were included based on their established associations with muscle strength and balance performance; neither reached statistical significance in any model (all *p* > 0.05) and are therefore not displayed in the tables. A diagonal covariance structure was used. Multicollinearity was assessed prior to modeling; all VIF values were below 2.5, indicating acceptable independence among predictors. Intrarater reliability for all strength measures was excellent (ICC(2,k) = 0.94–0.97), as classified according to Koo et al. [33]. All analyses were performed using SPSS 20.0 (IBM Corp., Armonk, NY, USA).

## 3. Results

A total of 104 participants were included in the analysis, of whom 74% were women and 26% were men (Table 1). Most participants reported the right leg as dominant (92.3%), while 7.7% reported left leg dominance.

The mean age of the sample was 38 years (range: 19–65 years), and descriptive anthropometric characteristics are presented in Table 2.

No significant differences were observed in YBT performance between the dominant and non-dominant leg across the anterior, posteromedial, and posterolateral reach directions or the composite score (Table 3). In contrast, small but statistically significant interlimb differences were observed in isometric hip strength. Hip abduction and external rotation strength were greater in the non-dominant leg, whereas hip extension strength was greater in the dominant leg.

Given the absence of interlimb differences in YBT performance, both limbs were included in subsequent analyses to maximize statistical power, with ipsilateral pairing of YBT and hip strength values (i.e., left YBT with left hip strength, right YBT with right hip strength). The dependency of repeated measurements within participants was accounted for using linear mixed models. Descriptive statistics for YBT performance and hip muscle strength, calculated across both limbs (208 observations), are presented in Table 4.

Correlation analyses, conducted across both limbs, revealed statistically significant positive associations between all hip strength measures and YBT performance (Table 5).

All observed correlations were of low magnitude (r = 0.30–0.49) for posterior reach directions and negligible for anterior reach (r = 0.19), according to the classification proposed by Mukaka [32]. The strongest bivariate association was observed between posterolateral reach and hip extension strength (r = 0.485).

Linear mixed model analyses (LMM) identified sex as the strongest and most consistent predictor of YBT performance across all reach directions and the composite score (Table 6).

Male participants achieved significantly higher scores than female participants across all directions (β = 7.8–8.9, *p* < 0.001-*p* = 0.725). Among hip strength measures, hip extension (β = 7.006, *p* = 0.033) and hip abduction (β = 11.447, *p* = 0.013) were significant independent predictors of posterolateral reach after controlling for sex, age, and BMI. Hip external rotation strength was a significant predictor of posteromedial reach (β = 11.429, *p* = 0.014). No hip strength measure independently predicted anterior reach or composite score when demographic covariates were included in the model (all *p* > 0.05, Table 7).

## 4. Discussion

The primary finding of this study is that sex was the strongest and most consistent independent predictor of YBT performance in healthy adults, with male participants achieving significantly higher scores across all reach directions. Among hip strength measures, hip extension and abduction strength were independently associated with posterolateral reach, and external rotation strength was independently associated with posteromedial reach, after controlling for sex, age, and BMI. No hip strength measure independently predicted anterior reach or composite score when demographic factors were considered. These findings suggest that demographic characteristics, particularly sex, substantially influence YBT performance and must be accounted for when interpreting hip strength–balance relationships. No meaningful differences in YBT performance were observed between dominant and non-dominant limbs, despite small but statistically significant interlimb differences in strength measures.

### 4.1. Sex as a Determinant of YBT Performance

The consistent and robust effect of sex on YBT performance across all reach directions warrants specific discussion. Male participants achieved higher YBT scores by approximately 8 points across directions, independent of hip strength and anthropometric variables. This finding is consistent with previous research by Miller et al. and Chimera et al., which documented sex-related differences in dynamic balance attributed to differences in lower-limb muscle mass, neuromuscular activation patterns, and biomechanical strategies during single-leg tasks [34,35].

Notably, females tend to exhibit greater knee valgus and hip adduction during single-leg loading tasks, which may constrain reach distances regardless of absolute hip strength levels, consistent with the findings of Crowell and colleagues [36].

It is also well established that males demonstrate significantly greater absolute hip extension and abduction strength than females, as highlighted by Vannatta et al. [37].

In this sample, the between-sex difference in muscle strength likely contributed independently to the observed differences in YBT performance. The unequal sex distribution (74% female) may have further amplified the observed sex effect, as the sex coefficient in the LMMs likely reflects not only neuromuscular and biomechanical differences between sexes, but also underlying strength differences that could not be fully separated in the present analyses. These findings should therefore be interpreted with caution, particularly when generalizing to populations with a more balanced or male-dominant sex distribution. Methodologically, the magnitude of the sex effect observed here underscores the importance of including sex as a covariate or conducting sex-stratified analyses in studies examining hip strength–balance relationships. The present findings suggest that the relationship between hip strength and YBT performance reported in unadjusted analyses is partly mediated or confounded by sex-related differences. Future studies with balanced sex representation or sex-stratified analyses are needed to isolate the independent contribution of hip strength to YBT performance within each sex. Clinicians and researchers interpreting YBT scores should therefore apply sex-specific reference values and exercise caution when comparing scores across mixed-sex samples without demographic adjustment.

### 4.2. Biomechanical Interpretation and Comparison with Previous Research

The independent association of hip extension and abduction strength with posterolateral reach, and of external rotation with posteromedial reach, is consistent with the biomechanical demands of these specific reaching tasks, particularly in the posteromedial and posterolateral directions, as highlighted in the research by Pinheiro and colleagues [38]. These tasks require precise control of the center of mass over a dynamically constrained base of support, placing substantial demands on posterior-chain musculature and sagittal-plane hip control [1,4,10]. During YBT execution, the stance limb must counteract forward trunk displacement and pelvic motion as reach distance increases. The hip extensors, primarily the gluteus maximus, are thought to play a critical role in generating the extension torque necessary to stabilize the pelvis and regulate trunk inclination. Greater hip extension capacity may be associated with more effective trunk–pelvis coupling, potentially allowing greater reach distances without compromising balance stability. Conversely, lower hip extensor strength has been associated with compensatory strategies, such as increased trunk flexion or reliance on distal joint control, which may be linked to reduced reach performance [3,13,16].

Using a prone testing position with the knee flexed to 90° likely enhanced the specificity of hip extension assessment by reducing hamstring contribution and isolating gluteal force production. This methodological approach strengthens the interpretation that the observed associations reflect true hip extensor capacity rather than generalized posterior-chain strength, which may explain why hip extension showed stronger associations with YBT outcomes than other hip muscle groups. In their study, Liu et al. examined the relationship between the gluteus maximus and hamstring muscles in various hip extensor strength testing positions [39].

Low correlations between hip abduction and external rotation strength and YBT performance observed in this study are consistent with previous reports [2,5,9]. These muscle groups primarily contribute to frontal and transverse plane stabilization of the pelvis and femur during single-leg tasks. The independent association of external rotation with posteromedial reach, and of abduction with posterolateral reach, suggests that these muscle groups are differentially associated with directional YBT performance. Their lack of independent association with anterior reach and composite score when demographic factors are controlled may reflect the dominant influence of sex-related differences in neuromuscular capacity and body composition on overall balance performance. This finding aligns with earlier interpretations of dynamic balance as a multi-joint, multi-planar task in which sagittal-plane hip control plays a central mechanical role [6,11,12].

The absence of meaningful limb dominance effects in YBT performance is consistent with the previous literature demonstrating symmetrical dynamic balance capacity in uninjured populations [7,14]. This suggests that, when neuromuscular function is intact, balance performance is governed more by global movement control strategies than by limb dominance.

#### 4.2.1. Practical and Clinical Implications

Although this study was conducted in a healthy population, the findings have practical implications for screening, training, and rehabilitation. The YBT is widely used to assess functional stability, identify individuals at increased injury risk, and monitor rehabilitation outcomes [15,17]. The present results indicate that hip extension and abduction strength are independently associated with posterolateral YBT performance, and that sex is a primary demographic factor influencing scores in all directions.

From a clinical practice perspective, clinicians should routinely record patient sex before interpreting YBT scores. Given the magnitude of the sex effect observed in this study (β = 7.8–8.9 points across all directions), applying sex-neutral reference values risks misclassifying female patients as having deficient balance when differences may largely reflect normal neuromuscular and biomechanical variation between sexes.

When posterolateral reach scores are reduced, clinicians should consider targeted assessment of hip extension and abduction strength, as these were identified as independent predictors of posterolateral performance in the present study. Conversely, reduced posteromedial reach scores may warrant specific evaluation of hip external rotation strength. This direction-specific interpretation allows clinicians to prioritize which muscle groups to assess and address, rather than applying a non-specific hip strengthening approach. In injury screening contexts, combining the YBT with a brief handheld dynamometry assessment of hip extension, abduction, and external rotation strength may improve the identification of individuals with modifiable strength deficits that may be associated with balance impairment. This is particularly relevant in sports medicine and physiotherapy settings where the YBT is already routinely used as a functional screening tool.

Regarding targeted training and rehabilitation, the association between hip extension and abduction strength and posterolateral reach performance suggests that posterior-chain strengthening exercises may be relevant for individuals with reduced posterolateral YBT performance. These exercises specifically target the gluteus maximus and gluteus medius, which were identified as independently associated with posterolateral reach in the present study. Similarly, the association between external rotation strength and posteromedial reach suggests that incorporating external rotator strengthening may be relevant for individuals with reduced posteromedial balance performance. Given that males demonstrated greater absolute hip extension and abduction strength than females in the present sample, sex-specific strengthening targets and progression criteria should be considered when designing rehabilitation and injury prevention programs.

For anterior reach performance, the weak and non-independent association with hip abduction strength (r = 0.194) has direct implications for rehabilitation and exercise programming. From a biomechanical perspective, anterior reach is primarily governed by sagittal-plane control of the stance limb, requiring adequate ankle dorsiflexion range of motion and quadriceps strength to allow forward displacement of the center of mass without loss of balance [12]. The limited association between hip abduction strength and this direction suggests that frontal-plane hip stabilization plays a secondary role in anterior reach performance in healthy adults. Consequently, rehabilitation programs targeting anterior reach improvement should prioritize ankle mobility work, such as weight-bearing lunge stretches and eccentric heel drops, along with quadriceps strengthening exercises, rather than focusing primarily on hip abductor training. For strength and conditioning practitioners, these findings suggest that anterior reach deficits identified during YBT screening should prompt assessment of ankle dorsiflexion and knee extensor capacity, rather than defaulting to a hip-focused intervention strategy.

However, given the cross-sectional design of this study, causal inference is not warranted. Prospective or experimental designs are needed to confirm whether targeted hip strengthening is associated with improved YBT performance in clinical and athletic populations.

#### 4.2.2. Strengths and Limitations

A major strength of this study is the use of strap-stabilized handheld dynamometry, which improves measurement reliability compared to unstabilized protocols and allows for more accurate estimation of joint torque. The sample size was larger than that of many comparable studies and included a broad adult age range, enhancing generalizability to the healthy adult population.

Several limitations should be acknowledged. The cross-sectional design precludes causal inference, and it cannot be determined whether greater hip extension strength is associated with improved balance or vice versa. Physical activity level, sport participation, and training history were not controlled and may have influenced both strength and balance performance. Additionally, only isometric strength was assessed; dynamic or eccentric measures of hip musculature may provide further insight into balance control mechanisms. Finally, the exclusive inclusion of healthy adults limits the direct applicability of these findings to clinical populations. It should also be noted that bivariate correlation analyses were performed across both limbs without correcting for within-participant dependency, as Pearson correlation does not account for the clustering of observations within individuals. Although this approach provides useful descriptive estimates of association, the effective sample size is smaller than the 208 observations used in the analysis, and the resulting correlation coefficients and confidence intervals should therefore be interpreted with caution. The linear mixed model analyses, which explicitly account for this dependency through the inclusion of participant as a random effect, are considered the primary analytical approach in this study.

Furthermore, the unequal sex distribution in the sample (74% female, 26% male) represents an important limitation that affects both the interpretation and generalizability of the present findings. Given that sex emerged as the strongest predictor of YBT performance, the predominance of female participants may have amplified the observed sex effect, and the findings may not be fully generalizable to populations with a more balanced or male-dominant sex distribution. Sex-stratified analyses were considered as an exploratory approach; however, with only 27 male participants, such analyses would have been underpowered and their results unreliable. Future studies should therefore recruit larger samples with balanced sex representation to enable robust sex-stratified analyses and to more precisely isolate the independent contribution of hip strength to YBT performance within each sex. Although the Kinvent dynamometer system has demonstrated high reliability for muscle strength assessment at other joints, direct validation data for hip-specific measurements using this device are currently limited in the literature. The intrarater reliability observed in the present study (ICC = 0.94–0.97) is, however, consistent with values reported for strap-stabilized handheld dynamometry at the hip using comparable devices.

Several additional limitations warrant acknowledgement. The demographic characterisation of participants was relatively superficial, and a number of contextual variables that may influence both hip strength and balance performance were not assessed or controlled. These include occupational physical demands, history of musculoskeletal injury, habitual exercise frequency and intensity, fat distribution and body composition beyond BMI, lower limb length asymmetry, pelvic width and morphology, and ethnicity. Each of these factors may independently or interactively influence YBT performance and hip muscle strength, and their omission limits the precision with which the observed associations can be attributed to hip strength per se. Future studies should incorporate a more comprehensive assessment of participant characteristics to enable more nuanced interpretation of hip strength–balance relationships across diverse populations.

## 5. Conclusions

Sex was the strongest and most consistent predictor of YBT performance in healthy adults, surpassing the independent association of hip strength across all reach directions. Among hip strength measures, hip extension and abduction strength were independently associated with posterolateral reach, while external rotation strength was independently associated with posteromedial reach after controlling for demographic variables. No hip strength measure independently predicted anterior reach or composite score. No meaningful limb dominance effects were observed.

These findings highlight the importance of accounting for sex when interpreting hip strength–balance relationships, as its omission risks overestimating the independent association of hip musculature with YBT performance. The directional specificity of hip strength associations suggests distinct mechanical relationships between posterior-chain and rotator muscles and single-leg balance tasks. Future prospective studies with balanced sex representation and dynamic strength assessments are needed to establish causal relationships and further inform clinical and athletic screening applications.

Future research should prioritise recruiting larger samples with balanced sex representation, or conduct sex-stratified analyses, to disentangle the independent contributions of sex and hip strength to YBT performance. Incorporating dynamic and eccentric assessments of hip muscle strength, alongside isometric measures, would provide a more comprehensive picture of the neuromuscular demands of single-leg balance tasks. Controlling for contextual variables such as habitual physical activity, sport participation, injury history, and body composition would further strengthen the interpretability of findings. Longitudinal and experimental designs are ultimately needed to establish causal relationships between hip strength and YBT performance, and to determine whether targeted strengthening interventions are associated with meaningful improvements in dynamic balance in both clinical and general populations.

## Figures and Tables

**Table 1:** Sex distribution and leg dominance of study participants (n = 104).

Variable	Category	n	%
Sex	Male	27	26
	Female	77	74
Dominant leg	Right	96	92.3
	Left	8	7.7

n: number of participants. %: percentage.

**Table 2:** Descriptive anthropometric characteristics of study participants (n = 104).

Variable	Min	Max	Mean ± SD
Age (years)	19	65	38.11 ± 15.92
Height (cm)	149	195	173.5 ± 9.7
Body mass (kg)	47	111	75.35 ± 15.26
BMI (kg/m^2^)	16	37	24.92 ± 4.09

BMI: Body Mass Index. SD: standard deviation.

**Table 3:** Comparison of Y-Balance Test performance and isometric hip strength between the dominant and non-dominant leg (n = 104).

Variable	Dominant Leg (M ± SD)	Non-Dominant Leg (M ± SD)	t	df	95%CI	*p*
Anterior reach (%)	75.19 ± 10.57	75.54 ± 10.85	−0.43	103	[−2.00, 1.30]	0.666
Posterolateral reach (%)	82.58 ± 13.95	82.24 ± 13.43	0.443	103	[−1.10, 1.78]	0.659
Posteromedial reach (%)	74.75 ± 14.23	75.47 ± 14.04	−0.99	103	[−2.17, 0.73]	0.324
Composite score (%)	81.32 ± 12.28	81.59 ± 12.19	−0.52	103	[−1.55, 1.01]	0.610
Hip abduction (Nm/kg)	0.63 ± 0.23	0.64 ± 0.24	−2.02	103	[−0.02, 0.00]	0.046 *
Hip external rotation (Nm/kg)	0.53 ± 0.23	0.57 ± 0.24	−3.45	103	[−0.06, −0.02]	<0.001 **
Hip extension (Nm/kg)	0.88 ± 0.37	0.83 ± 0.34	2.6	103	[0.01, 0.09]	0.01 *

M: mean; SD: standard deviation; t: t-value; df: degrees of freedom; CI: confidence interval for the difference between dominant and non-dominant leg (dominant–non-dominant); *p*: *p*-value. * *p* < 0.05; ** *p* < 0.001.

**Table 4:** Descriptive statistics for Y-Balance Test performance and isometric hip strength across both limbs (n = 208 observations from 104 participants).

Variable	Min	Max	Mean ± SD
Anterior reach (%)	47.51	110.71	75.36 ± 10.69
Posterolateral reach (%)	46.51	119.3	82.41 ± 13.7
Posteromedial reach (%)	37.86	113.49	75.11 ± 14.11
Composite score (%)	50	117	81.45 ± 12.21
Hip abduction (Nm/kg)	0.23	1.54	0.63 ± 0.24
Hip external rotation (Nm/kg)	0.05	1.44	0.55 ± 0.24
Hip extension (Nm/kg)	0	1.8	0.86 ± 0.36

SD: standard deviation. Values represent pooled descriptive statistics across both limbs (208 observations from 104 participants). Reach distances are normalised to leg length and expressed as a percentage.

**Table 5:** Pearson correlation coefficients between isometric hip strength measures and Y-Balance Test performance (n = 208 observations from 104 participants).

Variable	r	95% CI	*p*	Strength of Correlation
Anterior reach–Hip abduction	0.194	[0.06, 0.33]	0.005	Negligible
Anterior reach–Hip external rotation	0.303	[0.17, 0.43]	<0.001	Low
Anterior reach–Hip extension	0.302	[0.17, 0.43]	<0.001	Low
Posterolateral–Hip abduction	0.394	[0.27, 0.51]	<0.001	Low
Posterolateral–Hip external rotation	0.443	[0.32, 0.55]	<0.001	Low
Posterolateral–Hip extension	0.485	[0.37, 0.59]	<0.001	Low
Posteromedial–Hip abduction	0.354	[0.23, 0.47]	<0.001	Low
Posteromedial–Hip external rotation	0.424	[0.30, 0.54]	<0.001	Low
Posteromedial–Hip extension	0.451	[0.33, 0.56]	<0.001	Low
Composite–Hip abduction	0.347	[0.22, 0.47]	<0.001	Low
Composite–Hip external rotation	0.413	[0.29, 0.53]	<0.001	Low
Composite–Hip extension	0.444	[0.32, 0.56]	<0.001	Low

r: Pearson correlation coefficient; CI: confidence interval calculated using Fisher’s Z transformation; *p*: *p*-value. Correlation strength interpreted according to Mukaka [32]: negligible (r < 0.30), low (0.30 ≤ r < 0.50). Correlations were computed across both limbs without correction for within-participant dependency and should be interpreted as descriptive estimates only.

**Table 6:** Linear mixed model results for posterolateral and posteromedial reach.

Outcome	Predictor	β	SE	t	*p*	95% CI
Posterolateral reach	Sex	8.100	2.802	2.859	0.005	[2.46, 13.56]
	Hip extension	7.006	3.267	2.144	0.033	[0.56, 13.45]
	Hip abduction	11.447	4.576	2.502	0.013	[2.42, 20.47]
	Hip ext. rotation	5.208	4.619	1.128	0.261	ns
Posteromedial reach	Sex	8.109	3.023	2.683	0.008	[2.12, 14.09]
	Hip ext. rotation	11.429	4.611	2.479	0.014	[2.33, 20.53]
	Hip extension	2.250	3.265	0.689	0.491	ns
	Hip abduction	6.232	4.708	1.324	0.187	ns

β: regression coefficient; SE: standard error; t: t-value; *p*: *p*-value; CI: 95% confidence interval; ns: not significant. Sex coded as male = 1, female = 0. Age and BMI were included in all models as covariates but did not reach statistical significance in any model (all *p* > 0.05) and are therefore not displayed. Random effect: participant.

**Table 7:** Linear mixed model results for anterior reach and composite score.

Outcome	Predictor	β	SE	t	*p*	95% CI
Anterior reach	Sex	7.832	2.388	3.280	<0.001	[3.10, 12.56]
	Hip extension	2.016	3.067	0.657	0.512	[−4.04, 8.07]
	Hip abduction	2.949	4.175	0.706	0.481	[−5.33, 11.23]
	Hip ext. rotation	1.554	4.409	0.353	0.725	[−7.17, 10.28]
Composite score	Sex	8.870	2.550	3.480	<0.001	[3.83, 13.91]
	Hip extension	4.310	2.500	1.730	0.086	[−0.60, 9.22]
	Hip abduction	6.560	3.700	1.770	0.078	[−0.73, 13.85]
	Hip ext. rotation	4.830	3.490	1.380	0.168	[−2.07, 11.73]

β: regression coefficient; SE: standard error; t: t-value; *p*: *p*-value; CI: 95% confidence interval. Sex coded as male = 1, female = 0 (reference). Age and BMI were included in all models as covariates but did not reach statistical significance in any model (all *p* > 0.05) and are therefore not displayed. Random effect: participant. No hip strength measure reached statistical significance for anterior reach or composite score after demographic adjustment. Age and BMI were included in all models as covariates; neither reached statistical significance in any direction (all *p* > 0.05) and are therefore not displayed in the tables.

## Data Availability

Data are available from the corresponding author upon reasonable request.

## Abbreviations

The following abbreviations are used in this manuscript:

YBT	Y-Balance Test
LMM	linear mixed model
BMI	Body mass index

## References

[1] Gribble P.A., Hertel J., Plisky P. (2012). Using the Star Excursion Balance Test to Assess Dynamic Postural-Control Deficits and Outcomes in Lower Extremity Injury: A Literature and Systematic Review. J. Athl. Train..

[2] Plisky P.J., Rauh M.J., Kaminski T.W., Underwood F.B. (2006). Star Excursion Balance Test as a Predictor of Lower Extremity Injury in High School Basketball Players. J. Orthop. Sports Phys. Ther..

[3] Picot B., Terrier R., Forestier N., Fourchet F., McKeon P.O. (2021). The Star Excursion Balance Test: An Update Review and Practical Guidelines. Int. J. Athl. Ther. Train..

[4] Filipa A., Byrnes R., Paterno M.V., Myer G.D., Hewett T.E. (2010). Neuromuscular Training Improves Performance on the Star Excursion Balance Test in Young Female Athletes. J. Orthop. Sports Phys. Ther..

[5] Butler R.J., Southers C., Gorman P.P., Kiesel K.B., Plisky P.J. (2012). Differences in soccer players’ dynamic balance across levels of competition. J. Athl. Train..

[6] Gonell A.C., Romero J.A.P., Soler L.M. (2015). Relationship between the y balance test scores and soft tissue injury incidence in a soccer team. Int. J. Sports Phys. Ther..

[7] McCann R.S., Crossett I.D., Terada M., Kosik K.B., Bolding B.A., Gribble P.A. (2017). Hip strength and star excursion balance test deficits of patients with chronic ankle instability. J. Sci. Med. Sport.

[8] Wilson B.R., Robertson K.E., Burnham J.M., Yonz M.C., Ireland M.L., Noehren B. (2018). The Relationship Between Hip Strength and the Y Balance Test. J. Sport Rehabil..

[9] Lee D.K., Kim G.M., Ha S.M., Oh J.S. (2014). Correlation of the Y-Balance Test with Lower-limb Strength of Adult Women. J. Phys. Ther. Sci..

[10] López-Valenciano A., Ayala F., López-Elvira J.L., Barbado D., Vera-García F.J. (2016). Impact of dynamic balance and hip abductor strength on chronic ankle instability. Eur. J. Hum. Mov..

[11] Garima, Malhotra D., Kapoor G., Nuhmani S. (2024). Correlation between hip muscle strength and the lower quarter Y-balance test in athletes following anterior cruciate ligament reconstruction. J. Bodyw. Mov. Ther..

[12] Olszewski M., Zając B., Mika A., Golec J. (2024). Ankle dorsiflexion range of motion and hip abductor strength can predict Lower Quarter Y-Balance Test performance in healthy males. J. Bodyw. Mov. Ther..

[13] Domínguez-Navarro F., Benitez-Martínez J.C., Ricart-Luna B., Cotolí-Suárez P., Blasco-Igual J.M., Casaña-Granell J. (2022). Impact of hip abductor and adductor strength on dynamic balance and ankle biomechanics in young elite female basketball players. Sci. Rep..

[14] Nelson S., Wilson C.S., Becker J. (2021). Kinematic and Kinetic Predictors of Y-Balance Test Performance. Int. J. Sports Phys. Ther..

[15] Ahern L., Nicholson O., O’Sullivan D., McVeigh J.G. (2021). Effect of Functional Rehabilitation on Performance of the 450 Star Excursion Balance Test Among Recreational Athletes with Chronic Ankle Instability: A Systematic Review. Arch. Rehabil. Res. Clin. Transl..

[16] Lanza M.B., Arbuco B., Ryan A.S., Shipper A.G., Gray V.L., Addison O. (2022). Systematic Review of the Importance 453 of Hip Muscle Strength, Activation, and Structure in Balance and Mobility Tasks. Arch. Phys. Med. Rehabil..

[17] Bhanot K., Kaur N., Brody L.T., Bridges J., Berry D.C., Ode J.J. (2019). Hip and Trunk Muscle Activity During the Star 456 Excursion Balance Test in Healthy Adults. J. Sport Rehabil..

[18] Zheng J., Xu B., Xiong X., Chen J. (2025). Hip muscle strength in patients with chronic ankle instability: A systematic review and meta-analysis. Phys. Ther. Sport.

[19] Yeum W.J., Lee M.Y., Lee B.H. (2024). The Influence of Hip-Strengthening Program on Patients with Chronic Ankle Instability. Medicina.

[20] Rodrigues C.A.S., Albano T.R., Pereira Melo A.K., Azevedo Tavares M.L., Lima P.O.P., Almeida G.P.L. (2024). Y-Balance Test after ACL Reconstruction: The relationship with knee and hip muscle strength, ankle dorsiflexion range of motion and postural stability. J. Bodyw. Mov. Ther..

[21] Macadam P., Cronin J., Contreras B. (2015). An examination of the gluteal muscle activity associated with dynamic hip abduction and hip external rotation exercise: A systematic review. Int. J. Sports Phys. Ther..

[22] Chen B., Liu L., Bin Chen L., Cao X., Han P., Wang C., Qi Q. (2021). Concurrent Validity and Reliability of a Handheld Dynamometer in Measuring Isometric Shoulder Rotational Strength. J. Sport Rehabil..

[23] Kosti C., Tsoukos A., Pelekis I., Paschalis V., Terzis G., Bogdanis G.C. (2025). Isokinetic vs. Hand-Held Dynamometry for Assessing Knee Flexor and Extensor Strength in Athletes: Evaluating a Low-Cost Alternative across the Range of Motion. J. Hum. Kinet..

[24] Trybulski R., Więckowski J., Muracki J., Matuszczyk F., Gałęziok K., Wilk M., Kużdżał A. (2025). Reliability and reproducibility of the Kinvent K-push dynamometer for assessing quadriceps strength and force development in athletes and untrained individuals. Front. Physiol..

[25] Bohannon R.W. (1988). Make tests and break tests of elbow flexor muscle strength. Phys. Ther..

[26] Kelln B.M., McKeon P.O., Gontkof L.M., Hertel J. (2008). Hand-held dynamometry: Reliability of lower extremity muscle testing in healthy, physically active adults. J. Sport Rehabil..

[27] de Leva P. (1996). Adjustments to Zatsiorsky-Seluyanov’s segment inertia parameters. J. Biomech..

[28] Winter D.A. (2009). Biomechanics and Motor Control of Human Movement.

[29] Shaffer S.W., Teyhen D.S., Lorenson C.L., Warren R.L., Koreerat C.M., Straseske C.A., Childs J.D. (2013). Y-balance test: A reliability study involving multiple raters. Mil. Med..

[30] Plisky P.J., Gorman P.P., Butler R.J., Kiesel K.B., Underwood F.B., Elkins B. (2009). The reliability of an instrumented device for measuring components of the star excursion balance test. N. Am. J. Sports Phys. Ther..

[31] Faul F., Erdfelder E., Lang A.G., Buchner A. (2007). G*Power 3: A flexible statistical power analysis program for the social, behavioral, and biomedical sciences. Behav. Res. Methods.

[32] Mukaka M.M. (2012). Statistics corner: A guide to appropriate use of correlation coefficient in medical research. Malawi Med. J..

[33] Koo T.K., Li M.Y. (2016). A Guideline of Selecting and Reporting Intraclass Correlation Coefficients for Reliability Research. J. Chiropr. Med..

[34] Miller M.M., Trapp J.L., Post E.G., Trigsted S.M., McGuine T.A., Brooks M.A., Bell D.R. (2017). The Effects of Specialization and Sex on Anterior Y-Balance Performance in High School Athletes. Sports Health.

[35] Chimera N.J., Smith C.A., Warren M. (2015). Injury history, sex, and performance on the functional movement screen and Y balance test. J. Athl. Train..

[36] Crowell K.R., Nokes R.D., Cosby N.L. (2021). Weak Hip Strength Increases Dynamic Knee Valgus in Single-Leg Tasks of Collegiate Female Athletes. J. Sport Rehabil..

[37] Vannatta C.N., Kernozek T.W. (2021). Normative Measures of Hip Strength and Relation to Previous Injury in Collegiate Cross-Country Runners. J. Athl. Train..

[38] Pinheiro L.S.P., Ocarino J.M., Bittencourt N.F.N., Souza T.R., Souza Martins S.C., Bomtempo R.A.B., Resende R.A. (2020). Lower limb kinematics and hip extensors strengths are associated with performance of runners at high risk of injury during the modified Star Excursion Balance Test. Braz. J. Phys. Ther..

[39] Liu J., Teng H.L., Selkowitz D.M., Asavasopon S., Powers C.M. (2022). Influence of hip and knee positions on gluteus maximus and hamstrings contributions to hip extension torque production. Physiother. Theory Pract..

